# Involvement of Oral Microbiome in the Development of Oral Malignancy

**DOI:** 10.3390/cancers17040632

**Published:** 2025-02-13

**Authors:** Hitoshi Isono, Shintaro Nakajima, Satoshi Watanabe, Aya K. Takeda, Haruka Yoshii, Ami Shimoda, Hisao Yagishita, Kenji Mitsudo, Mitomu Kioi

**Affiliations:** 1 Department of Oral and Maxillofacial Surgery, Yokohama City University Graduate School of Medicine, Yokohama 236-0004, Japan; t166011g@yokohama-cu.ac.jp (H.I.); nakajima@caltech.edu (S.N.); yoshii.har.kl@yokohama-cu.ac.jp (H.Y.); t216030d@yokohama-cu.ac.jp (A.S.); mitsudo@yokohama-cu.ac.jp (K.M.); 2 Department of Life Science Dentistry, The Nippon Dental University, 1-9-20 Fujimi, Chiyoda-ku, Tokyo 102-8159, Japan; 3 Division of Biology and Biological Engineering, California Institute of Technology, Pasadena, CA 91125, USA; 4 Cykinso, Inc., 1-36-1 Yoyogi, Shibuya-ku, Tokyo 151-0053, Japan; s.watanabe@cykinso.co.jp (S.W.); takeda@cykinso.co.jp (A.K.T.); 5 Division of Oral Diagnosis, Dental and Maxillofacial Radiology and Oral Pathology Diagnostic Services, The Nippon Dental University Hospital, 1-9-20 Fujimi, Chiyoda-ku, Tokyo 102-8159, Japan; h-yagishita@tky.ndu.ac.jp

**Keywords:** oral squamous cell carcinoma, periodontitis, microbiota, 16S rRNA, *Porphyromonas gingivalis*

## Abstract

Objective: This study aimed to identify periodontal pathogens involved in the onset and progression of OSCC. Methods: Saliva samples were collected from 112 patients without oral mucosal diseases (OMDs) as controls; 36 patients with oral potentially malignant disorders (OPMDs); and 104 patients with OSCC. Periodontal examinations were performed on all patients. Endpoint PCR was performed for seven species of oral pathogens. The 16S rRNA analysis was performed using 20 DNA samples from each group. Results: Periodontitis tended to worsen in the OMDs group compared to the control group. The number of oral bacteria was significantly higher in the OSCC group than in the other groups. The detection rates of *P. gingivalis* and *Aggregatibacter actinomycetemcomitans* (*A. actinomycetemcomitans*) were significantly higher in the OSCC group than those in the control group. From 16S rRNA analysis, the relative abundance of *Prevotella buccae* and *intermedia* was significantly higher in OSCC than in the control. Moreover, LPS derived from *P. gingivalis* contributes to the early development of oral epithelial precancerous lesions and carcinomas in mice. Conclusions: Specific periodontal pathogens are present in the oral cavities of patients with OPMDs and OSCC, and changes in the bacterial flora due to their presence may contribute to the onset and progression of OMDs.

## Simple Summary

It has been reported that periodontitis may contribute to the onset and progression of oral squamous cell carcinoma (OSCC). However, it remains unclear which periodontal pathogens and virulence factors contribute to these diseases. This study aimed to identify the periodontal pathogens involved in the onset and progression of oral potentially malignant disorders and OSCC. We found that periodontitis worsened in OSCC, a higher proportion of OSCC patients had periodontal pathogens such as *Porphyromonas gingivalis (P. gingivalis*), and a particular bacterium occupied a high ratio in the oral microbiota. We also confirmed that lipopolysaccharide (LPS) derived from *P. gingivalis* promoted oral epithelial precancerous lesions and oral cancer in mice. The results of this study may lead to the development of an oral cancer prevention strategy by controlling bacterial infections, highlighting the importance of oral hygiene.

## 1. Introduction

Oral cancer is subcategorized as head and neck cancer, and oral squamous cell carcinoma (OSCC) accounts for more than 90% of all oral cancer [1]. The incidence of OSCC is increasing worldwide. In addition, the number of patients with OSCC is increasing with an aging population in Japan [2]. Despite advances in various treatments, including surgery, radiotherapy, and chemotherapy, the overall 5-year survival rate of patients is approximately 60–70%. Early diagnosis and optimal treatment can increase the success rate of OSCC treatment [3].

An association between cancer and chronic inflammation has been reported both epidemiologically and experimentally. In 1775, Pott reported that scrotal skin cancer was more common in chimney sweeps [4]. In the early 20th century, Yamagiwa and Ichikawa demonstrated that skin cancer is caused by chronic inflammation resulting from continuous rubbing of coal tar [5]. Furthermore, beginning with the reported association of *Helicobacter pylori* infection with the development of gastric cancer [6], it has become clear that chronic inflammation caused by pathogenic infections increases the risk of developing certain cancers [7,8,9]. Based on these findings, we hypothesized that periodontal disease, a chronic inflammatory disease caused by periodontal pathogen infections, may be a risk factor for various cancers, including OSCC. Our group previously reported that *Porphyromonas gingivalis* (*P. gingivalis*) in the oral cavity is significantly associated with non-alcoholic fatty liver disease, which is recognized as a precancerous lesion, and that periodontal treatment decreases *Fusobacterium nucleatum* (*F. nucleatum*) in the feces of patients with colorectal tumors [10,11]. Some case–control and cohort studies have shown a positive correlation between the prevalence of periodontal disease and OSCC [12]. Bacterial culture experiments have shown that periodontal pathogenic bacterial colonization is strongly correlated with gingival inflammation and cancer progression [13,14]. In addition, the ratio of anaerobic bacteria increased compared to that of aerobic bacteria on the surface of OSCC, and *P. gingivalis* and *Fusobacteria* were found to be more widespread [15,16]. Katz et al. reported that *P. gingivalis* can invade OSCC tissue [17]. These reports suggest that infections with specific periodontal pathogens may contribute to the development and progression of OSCC. Recently, various studies have used 16S rRNA sequencing with next-generation sequencing (NGS) to assess the oral microbiota profile of patients with cancer. For example, AI-Hebshi et al. showed that *Pseudomonas aeruginosa* and *F. nucleatum* were associated with OSCC development [18]. Another report indicated that periodontal pathogens were more abundant in OSCC lesions than in the controls [19]. In addition, it has been reported that the relative abundance (RA) of specific bacterial species increases and the oral microbial flora changes as OSCC progresses [20,21]. Based on these reports, the analysis and comprehension of the characteristic bacteria and microbiota in the oral cavity of patients with OSCC is extremely important because it may lead to the prevention and treatment of OSCC through bacterial infection control. However, it has not been attempted to detect characteristic periodontal pathogens in patients with OSCC via molecular biology and 16S rRNA methods. In addition, information on the characteristic periodontal pathogens of oral potentially malignant disorders (OPMDs) and OSCC patients is insufficient.

Thus, our study aimed to identify the candidate periodontal pathogens involved in the onset and progression of OSCC. We also performed the same analysis in patients with OPMDs to examine the same and different aspects of the detected periodontal pathogens and the composition of the oral microbiota, as in patients with OSCC. Finally, we investigated the effects of periodontal pathogens on the development of oral epithelial precursor lesions and oral cancer using a mouse model.

## 2. Materials and Methods

### 2.1. Study Subjects

In total, 252 patients were recruited for this study. Patients were classified into three groups (Table 1): control (112 controls), OPMDs (36 patients), and OSCC (104 patients). The inclusion criteria for patients with periodontitis were patients who had not received any periodontal treatments within the six months or any antibiotics within the three months and had at least 10 residual teeth. Periodontal examination was performed periodontal pocket depth (PD) and bleeding on probing (BOP) were assessed at the buccal site of each tooth using a periodontal pocket probe. The number of oral bacteria was counted by the back of the tongue swab samples using a rapid oral bacteria detection device (Bacterial Counter, DU-AA01NP-H; Panasonic Health Care, Tokyo, Japan) [22]. Saliva samples were collected before the initiation of the treatment. The controls were defined as individuals without any oral mucosal diseases (OMDs).

### 2.2. Saliva Collection and DNA Extraction

Patients gargled with 10 mL of sterile normal saline for 1 min and spat into a sterile tube. After collection, samples were centrifuged at 6000 rpm for 20 min, and the cellular parts were collected and stored at −80 ◦C until use. The pellet was resuspended with 0.5 mL of saline, and then lysed with 80 μL of nuclei lysis solution (A7941; Promega, Madison, WI, USA) at 80 °C for 5 min. After adding 60 μL of protein precipitation solution (A795A; Promega, Madison, WI, USA), the mixture was placed on ice for 5 min and then centrifuged at 15,000 rpm for 3 min. The supernatant was placed in a 1.5 mL tube, and DNA was purified by standard phenol–chloroform extraction followed by ethanol precipitation. DNA pellet was lysed in 50 μL Tris-EDTA buffer. The DNA quantity and quality were measured with a NanoDrop ND-1000 spectrophotometer (Thermo Fisher Scientific, Waltham, MA, USA).

### 2.3. Endpoint-Polymerase Chain Reaction (PCR) Assay

The PCR reaction was carried out in TaKaRa PCR Thermal Cycler MP (TP3000; TAKARA Bio, Shiga, Japan) with Quick Taq HS DyeMix (DTM-101; TOYOBO, Osaka, Japan) following the manufacturer’s instructions. Target bacteria, primer sequences, amplification conditions, and references are shown in Supplementary Table S1a,b. The final PCR product was evaluated with a 2% agarose gel stained by Midori Green Direct (NE-MG06; NIPPON Genetics, Tokyo, Japan).

### 2.4. 16S rRNA Sequencing and Analysis Using DNA Extracted from Mouth-Wash Saline

The V1-V2 region of the 16S rRNA gene was amplified using forward primer (16S_27Fmod: TCG TCG GCA GCG TCA GAT GTG TAT AAG AGA CAG AGR GTT TGA TYM TGG CTC AG) and reverse primer (16S_338R: GTC TCG TGG GCT CGG AGA TGT GTA TAA GAG ACA GTG CTG CCT CCC GTA GGA GT) with KAPA HiFi Hot Start Ready Mix (Kapa Biosystems, Wilmington, MA, USA). To sequence 16S amplicons by the Illumina MiSeq platform, dual index adapters were attached using the Nextera XT Index kit (Illumina, San Diego, CA, USA). Each library was diluted to 5 ng/μL, and equal volumes were mixed to 4 nM. The DNA concentration of the mixed libraries was quantified by qPCR with KAPA SYBR FAST qPCR Master Mix (KK4601, KAPA Biosystems) using primer 1 (AAT GAT ACG GCG ACC ACC) and primer 2 (CAA GCA GAA GAC GGC ATA CGA). The library preparations were carried out according to the 16S library preparation protocol of Illumina (Illumina). Libraries were sequenced using the MiSeq Reagent Kit v2 (500 Cycles) for 250 bp pair-ends (Illumina). Sequence files are available to show on request.

### 2.5. Taxonomy Assignment Based on the 16S rRNA Gene Sequence

Sequences were processed using the DADA2 plugin in QIIME 2 [23]. Reads were truncated at 230 bp (forward) and 220 bp (reverse) based on the quality profile. Low-quality reads were filtered using an expected error threshold of 2, and chimeric sequences were removed via the consensus method. The mean number of reads per sample after quality filtering was 39,477, with a minimum of 28,633 and a maximum of 60,894 reads per sample. A detailed summary of the sequencing depth, including the number of reads before and after filtering for each sample, is provided in Supplementary Table S2.

All data manipulation, analyses, and graphics were conducted using R and RStudio (versions 3.4.3 and 1.4.1100, respectively). The R package qiime2R [24] and microbiome R [25] were used for all analyses. The R package tidyMicro [26] and ggplot2 [27] were used for visualization. Research Cohort characteristics were summarized as frequencies (%) for categorical variables and means (standard deviations) or medians (interquartile range [IRQ]) for continuous variables. Differences in these characteristics were assessed using Fisher’s exact test for categorical variables and *t*-tests for continuous variables. RA was calculated as the number of sequenced reads for each taxon in a sample, standardized by the total number of sequences generated for each sample. A list of taxa and their RA in each sample are provided in Supplementary Table S3. Only taxa that were present in at least 1% of the cohort and had an RA of at least 0.01% in at least one sample were included in the analyses. Sequence counts for taxa that did not meet these requirements were aggregated into an “Other” category. These filtering requirements were applied at the species levels. Sequence counts that could not be classified at the taxonomic level of interest were left as unclassified counts of the lowest level possible. The alpha-diversity was evaluated at the ASV level using the Shannon index and Observed OTUs, and significant differences were evaluated using ANOVA. Beta-diversity was used to evaluate differences in the community composition between samples using the weighted and unweighted UniFrac distance method. Differences in Bray–Curtis distances between groups were assessed using a non-parametric permutation-based multivariate analysis of variation (PERMANOVA) test using the vegan package with 999 permutations [28]. We tested for significant impacts from the co-occurrences between disease groups, and each covariate (age, sex, number of teeth, PD, BOP, and number of oral bacteria) measure for this distance was measured by including interaction terms between the two phenotypes in each model. Principal coordinate ordination was performed using the cmdscale function [28]. To assess cross-sectional differences in the RAs of phylum-, genus-, and species-level taxa, we performed differential abundance analyses using a parametric or non-parametric *t*-test or ANOVA. We returned the *p*-values and Benjamin and Hochberg’s adjusted *p*-values as implemented in the ALDEx2 R package [26]. To test interaction effects between disease groups and each covariate measure, we evaluated using generalized linear models (GLM) assuming a negative binomial distribution and log link function; the total number of sequences was used as an offset [26]. For each taxon, a GLM model was first fit, including an interaction term between disease groups and each covariate measure. If the interaction term was significant (*p* < 0.05), it was determined that the effect of disease groups on the taxa was modified by each covariate measure, and results were reported for these models. If the interaction term was not significant, the interaction term was removed from the model, and the independent effect of disease groups, after adjusting for each covariate measure, was modeled and reported.

### 2.6. Mouse Oral Mucosal Lesion Formation Experiment

Four-week-old C57BL/6JJcl female mice (Japan CLEA, Tokyo, Japan) were obtained, housed at 5 per cage, provided with a regular chow diet, and maintained in a 12 h/12 h light/dark cycle. Mice were randomly divided into three groups (*n* = 9 mice per group): Control, 4-Nitroquinoline 1-oxide (4NQO), and 4NQO + lipopolysaccharide (LPS) of *P. gingivalis*. Mice in the control group were provided with regular water, and mice in the other two groups were administered regular water including 100 ppm 4NQO for 16 weeks. Mice in 4NQO + LPS group were repeatedly injected with 1.67 ppm LPS (14946-71; Invivogen, San Diego, CA, USA) at the palatal gingiva of the maxillary left first molar three times per week at the same time as the starting 4NQO administration. The injection was continued for 11 weeks after 4NQO withdrawal. The presence of oral mucosal diseases was observed and recorded weekly. Three mice were sacrificed on week 17, and all the remaining mice were sacrificed on week 27 in both groups. Their tongues were harvested and processed for histological examination.

### 2.7. Hematoxylin and Eosin Staining

Tongue samples were fixed with 4% paraformaldehyde–phosphate-buffered saline overnight at 4 °C. These were immersed in a 10, 20, and 30% sucrose solution and then embedded in OCT compound (Sakura Finetek). Frozen sections (7 μm) were stained with H&E and visualized with a Leica DM500 microscope and MC170 HD camera (Leica Microsystems, Wetzlar, Germany).

### 2.8. Micro-Computed Tomography (μCT) Analysis

The alveolar bone resorption of the maxilla was evaluated using a 3D micro X-ray CT imaging device (μCT) (R_mCT2, Rigakusha, Tokyo, Japan). A CT scan of the first molar on the left side of the maxilla was performed under the conditions of a tube voltage of 90 kV and a tube current of 160 μA. The alveolar bone resorption level was evaluated from the obtained three-dimensional CT image by measuring the position of the alveolar bone apex according to the method of Tokunaga et al. [29]. The distance (CEJ-ABC) between the cement–enamel junction (CEJ) and the alveolar bone crest (ABC) in the mesial region of the palate was measured. Micro-CT images were taken every two weeks, the distance between CEJ and ABC was measured, and the amount of bone resorption was evaluated.

### 2.9. Statistical Analysis

Clinical parameters (age and sex) and results of endpoint-PCR were analyzed by chi-square test. Residual analysis was used to identify cells contributing to the chi-square test results. The results of periodontal examinations (number of teeth, PD, BOP, and number of oral bacteria) were analyzed by the Mann–Whitney U test. *p*-values were adjusted using the Bonferroni correction method for the multiple comparisons problem. Two-sided probability values less than 0.05 were considered statistically significant.

## 3. Results and Discussion

### 3.1. Periodontal Disease Is Worse in OPMDs and OSCC Groups than in the Control Group

To investigate the severity of periodontal disease among the three groups, we statistically analyzed their clinical characteristics. Patient characteristics are summarized in Table 1 and Supplementary Figure S1. There was a significant difference in sex between the three groups. Residual analysis revealed that the proportion of males in the OSCC group was significantly higher than expected. Since the morbidity of OSCC is higher in males than in females [30,31], this result is consistent with previous reports. Regarding the clinical parameters, the number of teeth in the OPMDs and OSCC groups was significantly lower than that in the control group. Conversely, the BOP in the two groups was significantly higher than that in the control group. Furthermore, the number of oral bacteria was significantly higher in the OSCC group than that in the other groups. The median bacterial number in OSCC was approximately 1.9 times higher compared to that in the control, suggesting that a large number of bacteria adhered not only to the tongue surface, but also to the entire oral mucosa, including the tumor tissue. These results indicate that patients with oral premalignant disease and oral cancer have more severe periodontal disease than those without OMDs.

### 3.2. The Detection Rates of P. gingivalis, A. actinomycetemcomitans, and T. denticola Are Significantly Higher in OSCC

Because patients with OSCC show advanced periodontal disease, it is possible that particular periodontal pathogens are detected at a higher frequency. To compare the detection rates of certain oral bacteria reported to be involved in periodontal disease among the three groups, we performed endpoint PCR using DNA extracted from the saliva. Seven species of oral bacteria were detected by PCR, and the results are shown in Table 2. The primer sequences and PCR conditions are listed in Supplementary Table S1a,b, respectively. The chi-square test results showed a significant difference in the detection rates among the three groups, except for *Prevotella intermedia* (*P. intermedia*). Residual analysis revealed that the detection rates of *P. gingivalis, Aggregatibacter actinomycetemcomitans* (*A. actinomycetemcomitans*), and *Treponema denticola* (*T. denticola*) in the OSCC group were significantly higher than expected. It has been known that *P. gingivalis* and *T. denticola* not only constitute the red complex that is thought to be the etiology of periodontal disease but may also enhance OSCC cells migration, invasion, and tumorigenesis in mice body [32]. In addition, Inaba et al. reported that the gingipain proteases of *P. gingivalis* promote the invasion of OSCC cell lines by activating proMMP9 [33]. Together with these reports, it has been suggested that the periodontal pathogens detected at a high rate in OSCC are associated with their etiology and progression. In contrast, the detection rates of *Streptococcus mutans*, *F. nucleatum*, and *Tannerella forsythia* (*T. forsythia*) in the OSCC group were significantly lower than expected. *F. nucleatum* is a periodontal pathogen that is highly correlated with oral cancer [21,34,35,36] and colorectal cancer [37,38]. *T. forsythia* is a gram-negative anaerobic bacterium classified as a red complex with *P. gingivalis* and *T. denticola*.

*T. forsythia* is associated with OSCC, as operational taxonomic units (OTUs) of *Tannerella* Genus have been detected in DNA extracted from oral rinse samples of patients with OSCC stage IV [21]. Interestingly, Fan et al. reported that *P. gingivalis* and *A. actinomycetemcomitans* are associated with a higher risk of pancreatic cancer [39]. These findings suggested that specific bacterial species can cause multiple cancers. These conformities and differences between previous studies and the present study may be due to differences in the types of specimens, races, ethnic backgrounds, and lifestyles of the subjects recruited. However, further studies are required to confirm these differences.

### 3.3. The Bacterial Species Composing Oral Microbiota Among the Three Groups Are Different

As a different approach from endpoint PCR, which only detects the presence or absence of specific bacteria, to clarify and compare the oral microbiota of each group of patients and compare them, we performed 16S rRNA analysis. For the analysis, 20 samples were extracted from each group so that the clinical parameters did not deviate significantly from the population. The parameters of the extracted samples are summarized in Supplementary Table S4.

**Figure 1:**
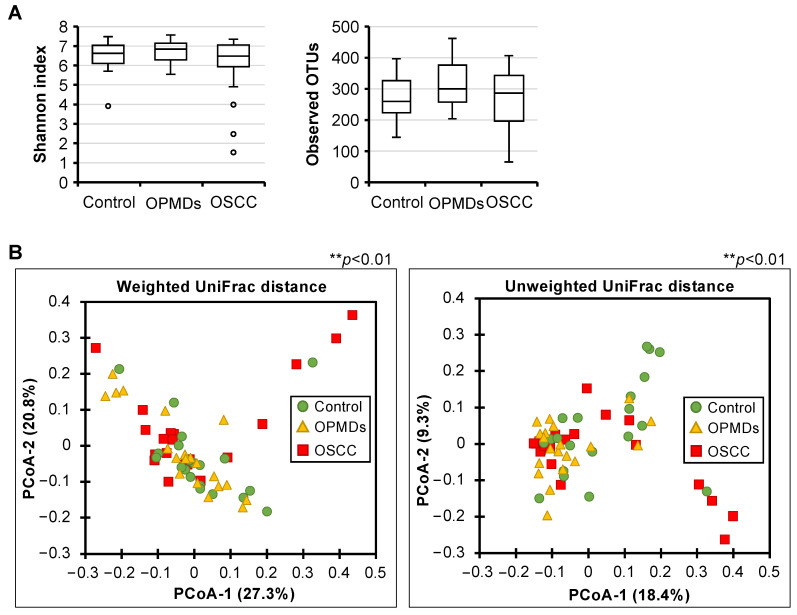
Alpha-diversity and beta-diversity analysis among three groups. Alpha-diversities are shown as box plots of Shannon index ((**A**) **left**) and Observed OTUs ((**A**) **right**). Beta-diversities are shown as weighted ((**B**) **left**) and unweighted ((**B**) **right**) UniFrac distance. PCoA plot, with respect to the bacterial abundance and composition. ** *p* < 0.01, Permutational MANOVA test.

First, we compared the alpha and beta diversities among the three groups. There were no differences in the alpha diversity of bacterial taxa among the three groups (Figure 1A). However, there were significant differences in beta diversity among the three groups in both weighted and unweighted UniFrac distances (Figure 1B). PERMANOVA revealed significant differences in beta diversity, although the magnitude of the differences was small (weighted UniFrac, *p* = 0.004; R2 = 7.5%; unweighted UniFrac, *p* = 0.004, R2 = 6.2%). These results suggest that there was no difference in the number of bacterial species in the oral microbiota of each group. However, there were differences in the abundance of common bacterial species, and there were specific bacterial species in each group.

To investigate the bacterial species contributing to the differences in oral microbiota among the three groups, we listed the bacterial species that showed significant differences in RA (Table 3).

When comparing the control and OSCC groups, *Prevotella buccae* and *P. intermedia* showed higher RA in OSCC than in the control, while *Actinomyces oris* showed lower RA. Interestingly, there was no significant difference in the detection rate of *P. intermedia* between the three groups in the PCR results (Table 2). This suggests that a high RA level in *P. intermedia*, but not its percentage, is associated with the onset of OSCC. The control and OPMDs groups *Filifactor alocis* (*F. alocis*) showed a higher RA in the OPMDs group than in the control group. *F. alocis* is a relatively new periodontal pathogen that has received increasing attention in recent years. It has been reported that it can interact with other common periodontal bacteria to form colonies of periodontal communities and potentially change the host cell proteome [40,41]. In addition, Yang et al. reported that the RA of *F. alocis* in patients with OSCC stage IV was significantly increased to more than 10-fold higher than that in healthy subjects [21]. The influence of *F. alocis* on the onset and progression of OPMDs and OSCC needs to be analyzed in further detail in future studies. Among the bacteria identified as genera, *Veillonella* species plural (spp.) showed lower RA in OPMDs and OSCC than in the controls. It has been shown that the RA of *Veillonella* spp. is elevated in the oral cavity of patients with good oral hygiene [42]. Furthermore, Yang et al. reported that the RA of *Veillonella parvula*, a species of *Veillonella* spp., was detected at a significantly higher rate in healthy controls than in patients with OSCC stage IV [21]. However, there are reports that *Veillonella* spp. have been detected in the oral microbiota of cancer patients, including OSCC patients with high RA [43,44,45]. Notably, the RA of *Oribacterium* spp. was lower, and that of *Parvimonas* spp. was higher in the OSCC group than in the other groups. *Oribacterium* spp. serve as indicators for distinguishing OSCC samples from healthy samples [46]. Previous studies have reported that *Parvimonas* spp. were enriched in tumor lesions, and the RA of this genus indicated significant differences between epithelial precursor lesions and the cancer group [19,47]. These results suggest that changes in the RA of these two genera (Oribacterium and Parvimonas) are strongly associated with the onset of OSCC. To the best of our knowledge, this is the first study to obtain data on bacterial genera and species with significant differences in RA between the control and OMDs (OPMDs and OSCC) groups.

### 3.4. The RA of P. gingivalis Is Higher in OSCC, Regardless of Age and the Number of Teeth

**Figure 2:**
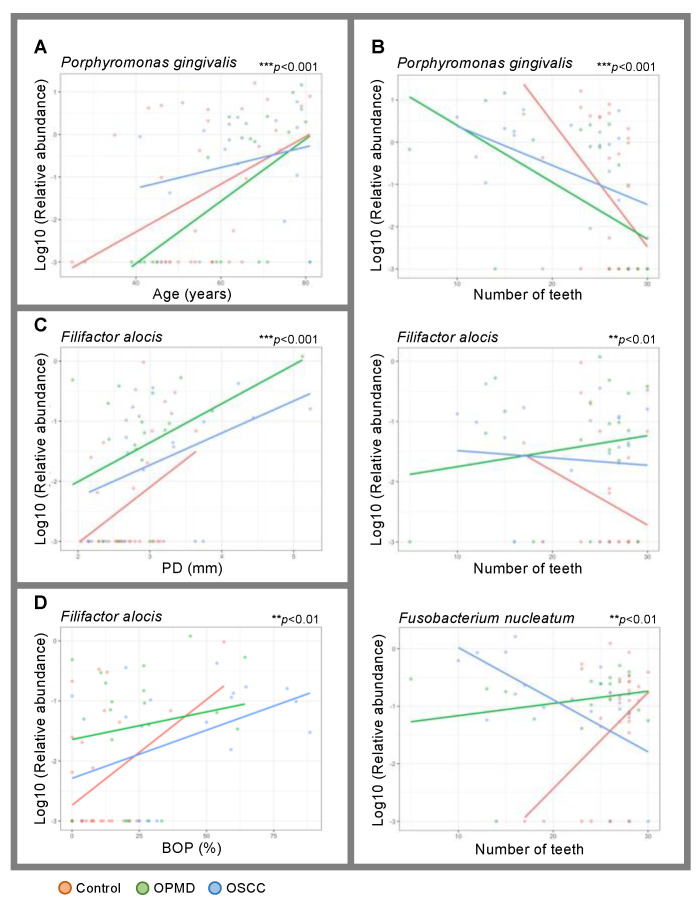
The graphs of linear mixed effects model. Each clinical parameter is used for the *x*-axis, and the relative abundance of specific bacterial spices (common logarithm) is used for the *y*-axis. The clinical parameter used for the *x*-axis is age (**A**), pocket depth (PD) (**B**), breeding on probing (BOP) (**C**), and the number of teeth (**D**), respectively. ** *p* < 0.01. *** *p* < 0.001.

To clarify the effect of each clinical parameter on the RA of the bacterial species, we visualized the relationship between them using a linear mixed-effects model. When age or number of teeth was plotted on the *x*-axis, there was a significant difference in the RA of *P. gingivalis* among the three groups (Figure 2A,B). The slope of the regression line for OSCC was more moderate than that for the other groups. This suggests that the RA of *P. gingivalis* is higher in OSCC, regardless of the patient’s age and number of teeth. When the number of teeth, PD, or BOP was plotted on the *x*-axis, there was a significant difference in the RA of *F. alocis* among the three groups (Figure 2B–D). The graphs show that The RA of *F. alocis* was higher in patients with fewer teeth, deeper PD, and higher BOP in the control group. This suggests a positive correlation between the worsening of periodontal disease and RA caused by *F. alocis* in the control group. Interestingly, the RA of *F. alocis* in OPMDs and OSCC was higher than that in the control group, regardless of the number of teeth, PD, or BOP. This result suggests that *F. alocis* is widespread throughout the oral cavity in the OMDs group and is not limited to sites of inflammation caused by periodontal disease. In addition, when the number of teeth was plotted on the *x*-axis, there was a significant difference in the RA of *F. nucleatum* among the three groups (Figure 2B). As shown in Figure 2B, the RA of *F. nucleatum* was higher in patients with more teeth in the control group. It is reported that *F. nucleatum* is present in proportion to CP malignancies of chronic periodontitis. Moreover, it is a locally increasing oral pathogen associated with periodontitis [48]. This result is inconsistent with those of previous reports because patients with milder periodontal disease are generally considered to have more teeth. This discrepancy should be investigated in future studies, along with quantitative PCR results. These results suggest that the high RA of *P. gingivalis* and *F. alocis* in the oral microbiota, regardless of the severity of periodontal disease, is associated with the presence of OSCC.

### 3.5. Periodontitis Induced by LPS of P. gingivalis Enhanced Carcinogenesis in 4NQO-Treated Mice

**Figure 3:**
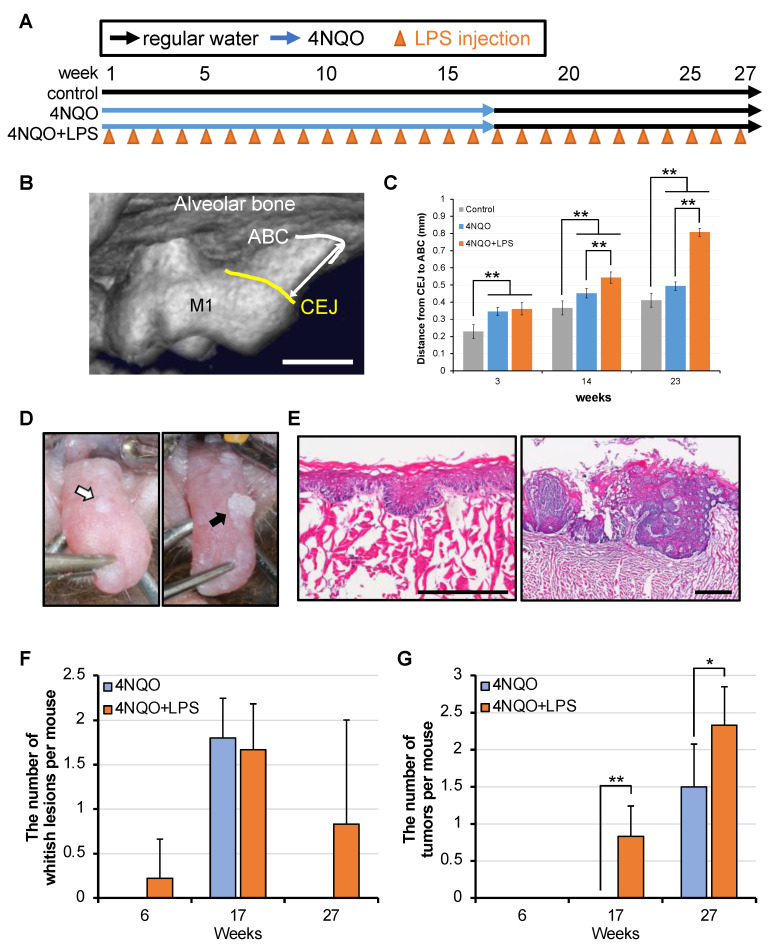
(**A**) Schematic diagram of mouse oral mucosal lesion formation experiment. (**B**) Micro-CT image of bone loss from cement–enamel junction (CEJ) to alveolar bone crest (ABC) of the maxillary left first molar. The measurement site is shown by the white arrow. The scale bar is 500 μm. (**C**) The mean ± SD of bone loss from CEJ to ABC. ** *p* < 0.01, Student’s *t*-test. *p*-values were adjusted using the Bonferroni correction method. n = 9 (3 weeks), 8 (14 weeks), and 6 (23 weeks). (**D**) Representative photographs of the back of the mouse tongue at 17 and 27 weeks after the start of the experiment in the 4NQO + LPS group. White and black arrows indicate tumor formation. (**E**) Representative photomicrographs of the hematoxylin-eosin (HE) staining of mouse tongue at 17 and 27 weeks after the start of the experiment in the 4NQO + LPS group. Scale bars are 500 μm. Changes over time in the number of whitish lesions (**F**) and tumors (**G**) per mouse. Graphs show the mean ± SD of the number of lesions per mouse in the corresponding week. * *p* < 0.05 and ** *p* < 0.01, Student’s *t*-test, compared with 4NQO.

Because the presence of *P. gingivalis* was associated with OSCC in this study and in various other reports, we investigated whether the oral microbiota promoted the early development of oral epithelial precursor lesions and oral cancer in mice. The experimental scheme is shown in Figure 3A. First, we examined the effects of *P. gingivalis* on periodontitis in mice. The bone loss was determined by micro-CT (Figure 3B), and the mean crestal bone loss on week 23 in 4NQO and 4NQO + LPS was 0.49 (±0.02) mm and 0.81 (±0.07) mm, respectively (Figure 3C). Bone loss was significantly more severe in the 4NQO + LPS group than that in the control and 4NQO groups. These findings suggest that the LPS injection of *P. gingivalis* is an effective approach to induce periodontitis in mice, which is consistent with previous reports [49]. Macroscopic findings indicated no tongue lesions in the control group. However, the 4NQO- and 4NQO + LPS-treated mice showed increased numbers of epithelial precursor lesions and cancerous masses. As shown in Figure 3F,G, early development of precursor lesions was observed in the 4NQO + LPS group; however, the number of precursor lesions was not significantly higher than that in the 4NQO group at weeks 17 and 27. This might be due to a reduction in the number of epithelial lesions that transitioned from a precursor status to a cancerous status. The number of cancerous masses was significantly higher in the 4NQO + LPS group than that in the 4NQO group at weeks 17 and 27. HE staining showed that the precursor lesions exhibited hyperplasia or dysplasia, and the cancerous masses exhibited carcinoma (Figure 3D,E). These results indicated that LPS from *P. gingivalis* contributes to the formation of tongue lesions induced by 4NQO.

### 3.6. Limitations of This Study and Future Challenges

We found that a higher proportion of OSCC patients had periodontal pathogens such as *P. gingivalis* and a particular bacterium occupied a high ratio in the oral microbiota. However, this study has some limitations. First, there is a possibility that regional bias has affected the results as the sample collection was carried out within a single center. Secondly, because the sample number in the OPMDs group was smaller than that in the other two groups, the results are more likely to be biased compared to the other two groups. Thirdly, because the groups were divided only based on the presence or absence of OMDs, the influences of other systemic diseases, past medical history, and preferences (e.g., smoking and alcohol drinking) on the results are not considered. Finally, further research is needed to clarify how the bacterial species identified in this study are involved in the onset and progression of OMDs, especially these bacterial functions in the oral microbiome maybe by targeting some specific bacteria.

## 4. Conclusions

**Figure 4:**
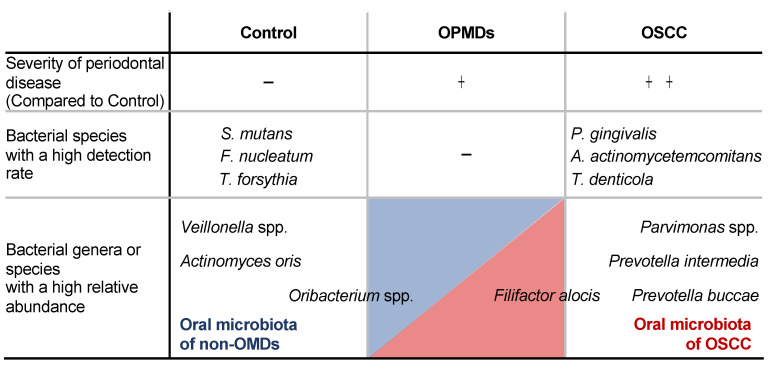
Schematic diagram of this study’s results. **Top line:** Statistical analysis of clinical parameters among the three groups showed that periodontal disease worsened with the progression of OMDs. **Middle line:** Endpoint-PCR results showed that the detection rates in the OSCC group of *P. gingivalis*, *A. actinomycetemcomitans*, and *T. denticola* were significantly higher than in the control group. **Bottom line:** The results of 16S rRNA analysis showed that *Actinomyces oris*, *Veillonella* spp. and *Oribacterium* spp. were presence with a highly relative abundance in the oral microbiota of control group patients, while *Prevotella intermedia* and *Parvimonas* spp. in the one of OSCC patients. These results suggest that changes in the oral microbiota due to the presence or absence of above oral bacteria may be involved in the onset and progression of OMDs.

The results of this study are summarized in Figure 4. In this study, we determined the changes in oral flora associated with the onset and progression of OSCC and identified the bacterial genera and species associated with OPMDs and OSCC. We also found that LPS from *P. gingivalis* promoted the early development of oral epithelial precursor lesions and oral cancer in mice. By identifying the characteristic periodontal pathogens and pathogenic factors directly involved in the onset and development of OPMDs and OSCC, we will be able to elucidate the direct relationship between periodontitis and the initiation of oral mucosal dysplasia and malignancy, leading to prevention strategies by controlling bacterial infections and affecting the oral microbiome, and highlighting the importance of oral hygiene.

## Figures and Tables

**Table 1:** The basic parameters of clinical samples.

Characteristics	Control	OPMDs	OSCC	*p*-Value ^1^
Number of patients	112	36	104	-
Age (years)				
Range	25–82	18–83	30–93	-
Mean (SD)	57.9 (14.6)	63.8 (14.5)	66.1 (13.4)	
Gender (%)				
Male	54 (48.2)	16 (44.4)	64 (61.5) ^▲^	*
Female	58 (51.8)	20 (55.6)	40 (38.5) ^▽^	
Number of teeth ^2^	28	25 ^††^	24.5 ^††^	-
PD (mm) ^3^	2.73	2.91	3.27	-
BOP (%) ^4^	4.80	21.98 ^††^	26.84 ^††^	-
Number of oral bacteria (cfu/mL) ^5^	0.98 × 10^7^	1.34 × 10^7^	1.86 × 10^7 ††,#^	-

^1^ * *p* < 0.05, chi-square test between three groups. ^▲^ *p* < 0.05, significantly high, ^▽^ *p* < 0.05, significantly low, residual analysis. *p*-values were adjusted using the Bonferroni correction method. ^2,3,4,5^: These values represent the medians. ^4^ Percentage of sites with BOP. ^††^ *p* < 0.01, Mann–Whitney U test, compared with control; ^#^ *p* < 0.05, Mann–Whitney U test, compared with OPMDs. *p*-values were adjusted using the Bonferroni correction method.

**Table 2:** Detection rate of target bacteria in three groups.

Target Bacteria	Detected or Not Detected	Control (n = 112)	OPMDs (n = 36)	OSCC (n = 104)	*p*-Value ^1^
		N (%)	N (%)	N (%)	
*S. mutans*	Detected	63 (56.3) ^▲^	11 (30.6)	34 (32.7) ^▽^	**
	Not detected	49 (43.7) ^▽^	25 (69.4)	70 (67.3) ^▲^	
*P. gingivalis*	Detected	54 (48.2) ^▽^	22 (61.1)	72 (69.2) ^▲^	**
	Not detected	58 (51.8) ^▲^	14 (38.9)	32 (30.8) ^▽^	
*P. intermedia*	Detected	45 (40.2)	14 (38.9)	55 (52.9)	ns
	Not detected	67 (59.8)	22 (61.1)	49 (47.1)	
*A. actinomycetemcomitans*	Detected	5 (4.5) ^▽^	0 (0) ^▽^	17 (16.3) ^▲^	**
	Not detected	107 (95.5) ^▲^	36 (100) ^▲^	87 (83.7) ^▽^	
*F. nucleatum*	Detected	104 (92.9) ^▲^	13 (36.1) ^▽^	61 (58.7) ^▽^	**
	Not detected	8 (7.1) ^▽^	23 (63.9) ^▲^	43 (41.3) ^▲^	
*T. forsythia*	Detected	94 (83.9) ^▲^	29 (80.6)	70 (67.3) ^▽^	*
	Not detected	18 (16.1) ^▽^	7 (19.4)	34 (32.7) ^▲^	
*T. denticola*	Detected	85 (75.9) ^▽^	31 (86.1)	93 (89.4) ^▲^	*
	Not detected	27 (24.1) ^▲^	5 (13.9)	11 (10.6) ^▽^	

^1^ * *p* < 0.05, ** *p* < 0.01, chi-square test between three groups. ^▲^ *p* < 0.05, significantly high, ^▽^ *p* < 0.05, significantly low, residual analysis. *p*-values were adjusted using the Bonferroni correction method. ns = not significant. The bacterial species composing oral microbiota among the three groups are different.

**Table 3:** Relative abundance at the species level by ALDEx2.

	Bacteria Name	wi.ep ^1^	wi.eBH ^2^	Effect
Control vs. OSCC	*Actinomyces*	0.001	0.044	−0.728
	***Actinomyces oris***	0.015	0.169	−0.656
	*Corynebacterium*	0.006	0.123	−0.528
	*Rothia*	<0.001	0.015	−0.804
	*Pseudopropionibacterium*	0.002	0.061	−0.644
	***Prevotella buccae***	0.006	0.117	0.502
	***Prevotella intermedia***	0.004	0.091	0.676
	*Oribacterium*	<0.001	0.026	−0.772
	*Parvimonas*	0.001	0.054	0.564
	*Veillonella*	0.001	0.043	−0.738
	*Leptotrichia*	0.001	0.039	−0.66
	*Kingella*	<0.001	0.023	−0.849
Control vs. OPMDs	*Streptococcus*	0.004	0.445	−0.569
	***Filifactor alocis***	0.049	0.566	0.512
	*Veillonella*	0.009	0.479	−0.508
OPMDs vs. OSCC	*Alloprevotella*	0.003	0.175	0.694
	*Oribacterium*	0.009	0.282	−0.542
	*Stomatobaculum*	0.001	0.111	−0.702
	*Parvimonas*	0.001	0.107	0.721
	*Lautropia*	0.003	0.176	−0.517

^1^ wi.ep: expected *p*-value of Mann–Whitney *U* test; ^2^ wi.eBH: expected Benjamini–Hochberg corrected *p*-value; **Bold**: Bacterial species revealed to species-level; Blue-filled: Bacterial species with significantly lower abundance relative to the group to compare; Red-filled: Bacterial species with significantly higher abundance relative to the group to compare.

## Data Availability

The raw data supporting the conclusions of this article will be made available by the authors on request.

## Supplementary Materials

The following supporting information can be downloaded at: https://www.mdpi.com/article/10.3390/cancers17040632/s1, Supplementary Table S1a: Species-specific primer sets for competitive PCR analysis; Supplementary Table S1b: Species-specific amplification conditions for PCR analysis; Supplemental Figure S1: Clinical periodontal parameters. Box plots of Number of teeth present (A), probing depth (PD) (B), breeding on probing (BOP) (C), and Number of oral bacteria (D) are shown. ^††^ *p* < 0.01, Mann–Whitney *U* test, compared with control; ^#^ *p* < 0.05, Mann–Whitney U test, compared with OPMDs. *p*-values were adjusted using the Bonferroni correction method; Supplementary Table S2: A detailed summary of the sequencing depth; Supplementary Table S3: List of taxa and their RA in each sample; Supplementary Table S4: Clinical characteristics of the study population for NGS. Refs. [50,51,52,53,54,55,56] are cited in Supplementary Materials.

## Abbreviations

The following abbreviations are used in this manuscript: oral squamous cell carcinoma (OSCC); lipopolysaccharide (LPS); oral mucosal diseases (OMDs); oral potentially malignant disorders (OPMDs); next-generation sequencing (NGS); relative abundance (RA); pocket depth (PD); bleeding on probing (BOP); 4-Nitroquinoline 1-oxide (4NQO).
